# Cardiac energy metabolism is decreased in male volunteers with prediabetes and does not normalize during the day

**DOI:** 10.14814/phy2.70242

**Published:** 2025-02-27

**Authors:** Vera H. W. de Wit‐Verheggen, Jakob Wefers, Carlijn M. E. Remie, Patrick Schrauwen, Vera B. Schrauwen‐Hinderling, Tineke van de Weijer

**Affiliations:** ^1^ Department of Nutrition and Movement Sciences, School for Nutrition and Translational Research in Metabolism Maastricht University Medical Center Maastricht The Netherlands; ^2^ Institute for Clinical Diabetology, German Diabetes Center Leibniz Institute for Diabetes Research at Heinrich Heine University Düsseldorf Düsseldorf Germany; ^3^ Clinical Epidemiology Leiden University Medical Center Leiden The Netherlands; ^4^ Department of Radiology and Nuclear Medicine Maastricht University Medical Center Maastricht The Netherlands

**Keywords:** cardiac energy status, PCr/ATP, prediabetes

## Abstract

Type 2 diabetes mellitus is characterized by a low cardiac energy status (PCr/ATP ratio), but it is unknown whether this also applies to prediabetes. Since PCr/ATP is correlated with elevated free fatty acids (FFA), a potentially lower PCr/ATP might be secondary to elevated FFA. To investigate this, we determined PCr/ATP and FFA levels in volunteers with prediabetes at two time‐points during the day. Eight male volunteers with prediabetes underwent a MRI/MRS scan to determine left ventricular ejection fraction (LVEF) and PCr/ATP ratio at 7 am and at 5 pm. For reference, these results were compared to eight non‐insulin resistant overweight or obese volunteers. Myocardial energy status was lower in the volunteers with prediabetes (PCr/ATP 1.03 ± 0.08) compared to non‐insulin resistant overweight or obese volunteers (PCr/ATP 1.22 ± 0.04, *p* < 0.05), but FFA were not significantly different between groups. LVEF was similar in the volunteers with prediabetes compared to healthy overweight and obese volunteers (*p* = 0.23). Volunteers with prediabetes have a lower myocardial energy status in the morning compared to healthy overweight and obese volunteers, while cardiac function remained normal. In addition, no differences between morning and evening measurements of cardiac energy status and function were found.

## 1 BACKGROUND

It has been recognized that patients with type 2 diabetes mellitus (T2DM) have a reduced cardiac energy status compared to healthy controls (Bugger & Abel, 2010; Diamant et al., 2003; Levelt et al., 2016; Scheuermann‐Freestone et al., 2003). This cardiac energy status, which is measured as the ratio of phosphocreatine (PCr) over adenosine triphosphate (ATP) by phosphorus magnetic resonance spectroscopy (^31^P‐MRS), was reported to be of prognostic value in heart failure (Neubauer et al., 1997). Considering that the risk for cardiovascular disease is already increased in prediabetes, the question arises whether a reduced cardiac energy status is also present in prediabetes.

In addition, PCr/ATP ratio was shown to correlate negatively with fasting plasma free fatty acid (FFA) concentrations, both in T2DM and in healthy control subjects (Bilet et al., 2011; Scheuermann‐Freestone et al., 2003). Therefore, the increase in circulating FFA in T2DM may be an underlying reason for decreased PCr/ATP. Also, in prediabetes FFA are elevated (Wefers et al., 2020), making a lower PCr/ATP ratio in prediabetes plausible. In addition, Ith et al. showed in healthy individuals a linear correlation between the cardiac lipid storage and FFA plasma concentrations; with high cardiac lipid content in the morning in fasted conditions, and low cardiac lipid storage in the evening in the postabsorptive state (Ith et al., 2014). Therefore, in line with the notion that FFA uptake in the heart is largely determined by FFA plasma concentrations, the heart is expected to rely more strongly on fat oxidation in the fasted state in the morning when FFA concentrations are high. As fat oxidation is less efficient in terms of ATP production relative to oxygen use, this may result in a decreased PCr/ATP ratio in the fasted state. The investigation of PCr/ATP in the morning and again later in the day may reveal whether elevated FFA are underlying a potentially decreased PCr/ATP in prediabetes.

In this study, we measured PCr/ATP, cardiac function, and FFA in volunteers with prediabetes and in overweight or obese controls. We examined whether PCr/ATP was lower in prediabetes and whether it normalized later in the day, together with normalizing FFAs. Therefore, we hypothesize that the individuals with prediabetes have a lower PCr/ATP ratio in the morning compared to their overweight or obese controls. Furthermore, we expect that PCr/ATP correlates with FFA and left ventricular ejection fraction (LVEF).

## 2 METHODS

### 2.1 Volunteers and study conditions

Eight male volunteers with overweight (BMI 25–35 kg/m^2^) and prediabetes between the ages of 40–75 years were recruited through advertisements in the vicinity of Maastricht. The study was conducted in accordance with the principles of the declaration of Helsinki and approved by the Ethics Committee of the Maastricht University Medical Center. All volunteers provided written informed consent. The study was registered at https://clinicaltrials.gov with identifier NCT03733743 (07/11/2018).

The in‐ and exclusion criteria and study protocol were described in detail previously (Wefers et al., 2020). Prediabetic participants had to fulfill at least one of four criteria in order to be included in the study: (1) impaired fasting glucose (IFG) 6.1 mmol/L‐6.9 mmol/L; (2) impaired glucose tolerance (IGT) 7.8 mmol/L‐11.1 mmol/L 2 h after 75 g glucose consumption; (3) HbA_1c_ of 5.7%–6.4%; or (4) low insulin sensitivity defined as glucose clearance rate ≤360 mL/kg/min according to the oral glucose insulin sensitivity (OGIS) model. By means of the Morningness‐Eveningness Questionnaire Self‐Assessment Version 1.3 (MEQ‐SA; Score between: 35 and 70) we excluded extreme morning or evening types. After a 7‐day run‐in period wherein volunteers adhered to a fixed lifestyle with a fixed diet, meal times, and sleeping times, they were admitted to the research unit at 12 pm on test day 1. They stayed overnight to standardize and monitor meals, physical activity, and bedtime; mimicking a real‐life situation. Magnetic resonance imaging (MRI) and MRS scans were performed on the first and second test day. Meals were provided at 8 am, 1 pm, and 6 pm in our research facilities. Blood drawings were performed after the MRI scans at 6 pm and 8 am and results here from were published previously (Wefers et al., 2020).

For reference, eight healthy non‐insulin resistant overweight and obese volunteers from an earlier study are included for comparison in the results (they all had a fasted glucose <6.0 mmol/L and a M‐value above 5.5 mg/kg/min measured with a 2‐step hyperinsulinemic euglycemic clamp, indicating normal insulin sensitivity (Roden, 2007)). In the control group, the MRS scan and blood drawings were performed under the same standardized conditions as in our volunteers with prediabetes, both groups were scanned at 7 am in fasted condition following an overnight stay in the respiration chamber, according the same scanning protocol. The in‐ and exclusion criteria and study protocol from these healthy overweight and obese volunteers were described in detail previously (Remie et al., 2020).

### 2.2 ^31^P‐MRS acquisition

**FIGURE 1:**
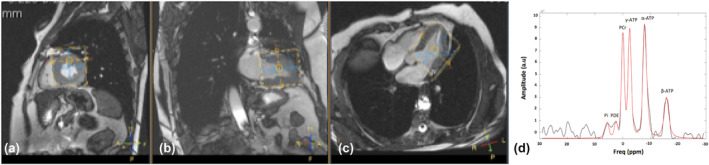
Planning of the volume of interest and cardiac ^31^P‐MRS spectrum. The volume of interest (yellow) is carefully placed in maximum systole to ensure the myocardium of the left ventricle is taken into account, without muscle mass of the pectoralis and diaphragm, minimizing voxel placement in the lungs. (a) sagittal view, (b) coronal view, (c) transversal view. In (d) Example of a MATLAB output of a cardiac ^31^P‐MRS spectrum; in black the original cardiac ^31^P spectrum with in red the by Mat lab created fit of the high energy phosphorus metabolite peaks Pi, PDE, PCR, γ‐ATP, and α‐ATP, at their typical resonance frequency.

In volunteers with prediabetes, cardiac energy status was determined on the first test day after a standardized lunch followed by a 4 h fast at 5 pm and on the second test day after an overnight fast at 7 am. In obese volunteers, measurements were only performed at 7 am. All measurements were performed on a 3.0T whole body MRI scanner (Achieva Tx, Philips Healthcare, Best, The Netherlands) with identical protocols. The myocardial PCr to ATP ratio was quantified by phosphorus magnetic resonance spectroscopy (^31^P‐MRS), using a 3D ISIS sequence, according to the protocol of Lamb et al. (1996). Volunteers were positioned prone and head first in the MRI scanner. A double tuned ^1^H and ^31^P surface cardiac coil (Rapid Biomedical, Rimpar, Germany) was placed under the volunteer's chest. After making scout‐images of the heart, the volume of interest was carefully placed over the total volume of the left ventricle of the heart (Figure 1a–c). Spectra were acquired in expiration during the end‐systolic phase, using a 90 degree hyperbolic secant pulse (NSA = 128 (16 full ISIS acquisitions), number of points = 2048, bandwidth = 3000 Hz, no frequency offset) with a repetition time of approximately 6 s. As the acquisition was triggered to the systolic phase, based on ECG‐triggering, the repition time (TR) needed to be defined in number of heart beats. Based on the heart rate, the number of heart beats closest to 6 s was calculated for each volunteer (if heart rate was below 55 bpm, TR = 5 heart beats was used, if heart rate was between 55 and 65 bpm, TR = 6 heart beats was used, if heart rate was 66–75 bpm, TR = 7 heart beats was used). The variation in repetition time was therefore minimized. For T1 correction, the actual TR was calculated individually for every subject based on the set TR (in heart beats) and the heart rate during the measurement and data were corrected for T1‐relaxation according to El‐Sharkawy et al. (2009). For example of a spectrum, see Figure 1d. Volunteers were continuously instructed (via the intercom) to breathe in the rhythm of the measurements, making sure to be in the expired position during acquisition.

### 2.3 ^31^P‐MRS spectral post‐processing

PCr and ATP resonances were quantified using a custom written MATLAB (MATLAB 2014b, The MathWorks, Inc., Natick, Massachusetts, United States) script. Values were corrected for T1 saturation (T1 was assumed to be 5.8 s for PCr and 3.1 s for gamma‐ATP according to El‐Sharkawy et al. (2009)) and expressed as ratio of PCr over gamma‐ATP (PCr/ATP). Spectral quality was similar in the two groups with similar signal‐to‐noise‐ratios.

### 2.4 Cardiac function with MRI

A single slice multiphase short axis image and a long axis image planned midventricular in the left ventricle of the heart were obtained in eight out of nine volunteers at both time‐points (5 pm and 7 am). Using dedicated software (OsiriX DICOM Viewer) end diastolic volume (EDV) and end systolic volume (ESV) were estimated according to the Hemisphere Cylinder Model, as described earlier (van de Weijer et al., 2012). From the EDV and ESV the LVEF and stroke volume (SV) were calculated.

### 2.5 Statistics

Data are presented as mean ± SD (standard deviation) unless indicated otherwise. Primary outcome measures of the study were cardiac PCr/ ATP ratio at 7 am and 5 pm, as determined by ^31^P‐MRS. Secondary outcome parameters were LVEF at 7 am and 5 pm, as determined by MRI. Statistical analyses were performed with the use of IBM Statistical Package for Social Sciences for MAC, version 23 (SPSS, Inc.). The effect of time on outcome variables was analyzed by a paired *t*‐test. Statistical significance was defined as a *p* value <0.05.

## 3 RESULTS

### 3.1 Volunteer characteristics

Eight male volunteers with a mean age of 67 ± 6 years who were overweight (BMI: 29.7 ± 1.9 kg/m^2^) participated in the study. Volunteer characteristics are summarized in Table 1. The characteristics of the eight healthy non‐insulin resistant overweight and obese volunteers from our earlier study, which are used for reference in the results, are as far as determined, displayed in Table 1.

**TABLE 1:** Volunteer characteristics.

Parameter	Prediabetes (*n* = 9)	Overweight or obese (*n* = 10)	*p* Value
Age (years)	67 ± 7	60 ± 3	0.031
BMI (kg/m^2^)	29.9 ± 1.9	29.6 ± 2.1	0.783
Fasting plasma glucose (mmol/L)	5.8 ± 0.5	5.2 ± 0.5	0.036
Fasting plasma insulin (μIU/mL)	12.7 ± 4.4	10.5 ± 6.8	0.46
QUICKI‐index	0.56 ± 0.06	0.62 ± 0.10	0.099
2‐h plasma glucose (mmol/L)	7.20 ± 1.0	NA	
HbA_1C_ (%)	5.3 ± 0.3	NA	
Glucose clearance (mL/kg/min)	337 ± 24	NA	

*Note*: Volunteers's characteristics at baseline. Values are depicted as mean ± standard deviation (*n* = 9). The data of the healthy volunteer group were derived from the previous study of Remie et al. (2020) and used as a reference for the data in this study.

Abbreviation: NA, not available.

### 3.2 Lower cardiac energy status in prediabetes

**FIGURE 2:**
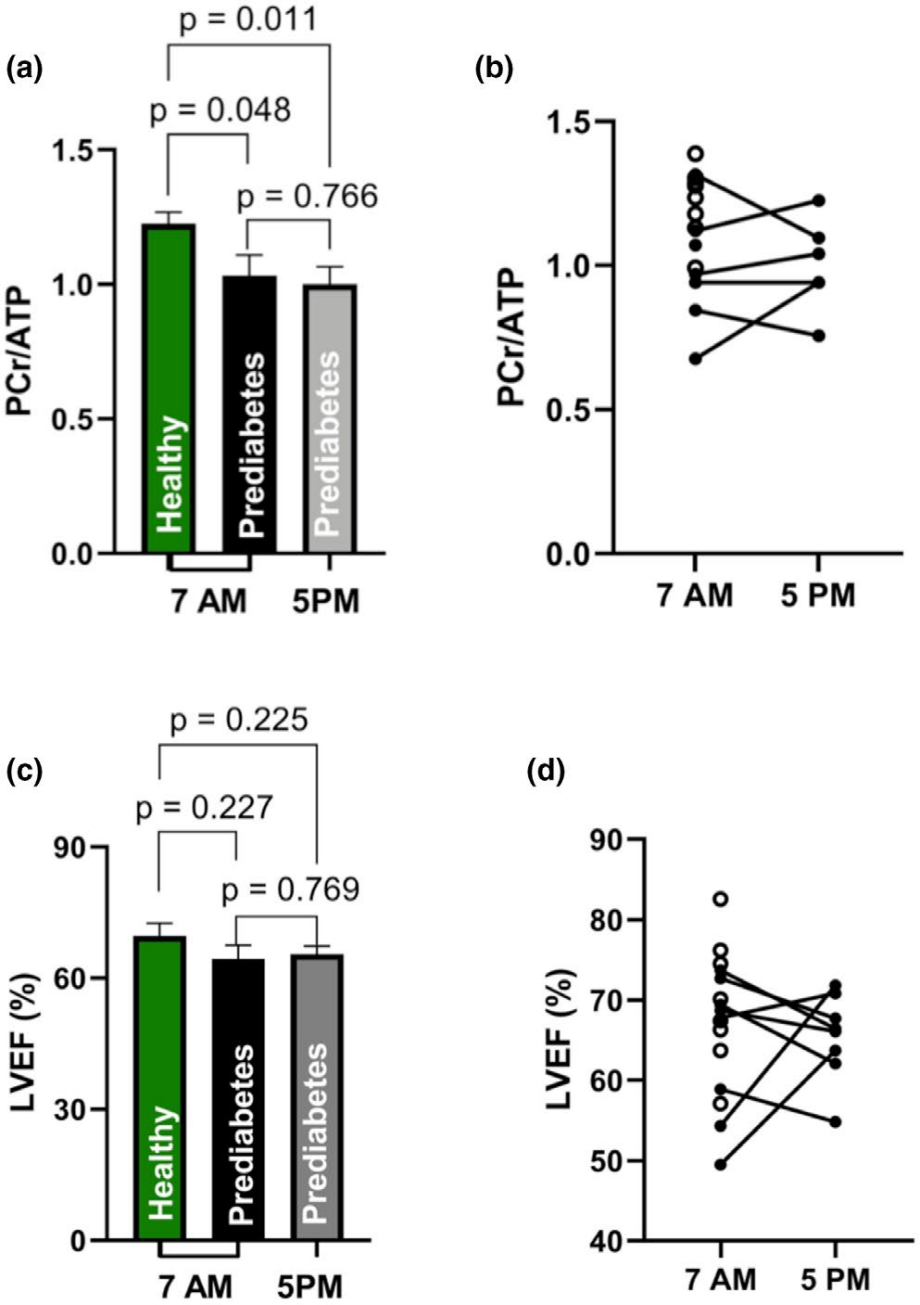
PCr/ATP ratio and LVEF at 7 am and 5 pm. (a) shows the PCr/ATP ratios at 7 am in the morning in eight healthy overweight and obese volunteers from the previous study (Remie et al., 2020), and the PCr/ATP ratio's in volunteers with prediabetes (*n* = 8) at 7 am and 5 pm. The PCr/ATP ratios in the obese controls (1.22 ± 0.04) differed significantly from the eight volunteers with prediabetes (1.03 ± 0.08, *p* < 0.05). There was no significant difference between the average PCr/ATP ratio in the morning (1.03 ± 0.08) and evening (1.00 ± 0.07) in the volunteers with prediabetes. (b) Shows the individual PCr/ATP ratio's for the volunteers with prediabetes at 7 am and 5 pm. (c) Shows LVEF in obese controls at 7 am (form the previous published study (Remie et al., 2020)) and volunteers with prediabetes at 7 am and 5 pm. Here, no differences in LVEF was observed between the healthy (71 ± 3%) and the volunteers with prediabetes (64 ± 3%, *p* = 0.23). In addition, no day‐night fluctuations in LVEF can be observed with LVEF 64 ± 3% in the morning and 65 ± 2% in the evening (*p* = 0.77). (d) Shows the individual LVEF data of the volunteers with prediabetes at 7 am and 5 pm. Values are depicted as mean ± standard error of the mean. Healthy controls are in (a) and (c) depicted in green and in (b) and (d) as white points. Values are depicted as mean ± standard error of the mean. The data of the healthy volunteer group were derived from the previous study of Remie et al. (2020) and used as a reference for the data in this study.

The PCr/ATP ratio in the morning in the volunteers with prediabetes (1.03 ± 0.08) was significantly lower than in the 10 healthy overweight and obese volunteers (1.22 ± 0.04) from our earlier study (Figure 2a,b, *p* = 0.04) (Remie et al., 2020).

### 3.3 No difference between fasted and fed cardiac energy metabolism

Due to technical difficulties, the spectra could not be acquired in two subjects in the afternoon. In the six subjects with prediabetes wherein we were able to measure both, morning and afternoon, the average PCr/ATP ratio in the evening measured at 5 pm was 1.00 ± 0.07. There was no significant difference between the average PCr/ATP ratio in the morning and in the evening (*p* = 0.77, Figure 2a,b). Heart rate was measured during the MRI scans and was not significantly different between the morning and late afternoon measurements (62.6 ± 9.6 vs. 60.3 ± 6.8, *p* = 0.15, respectively), it was also not different between controls and individuals with prediabetes (63.3 ± 6.9 vs. 62.6 ± 9.6, *p* = 0.84).

### 3.4 Plasma free fatty acid concentrations

**FIGURE 3:**
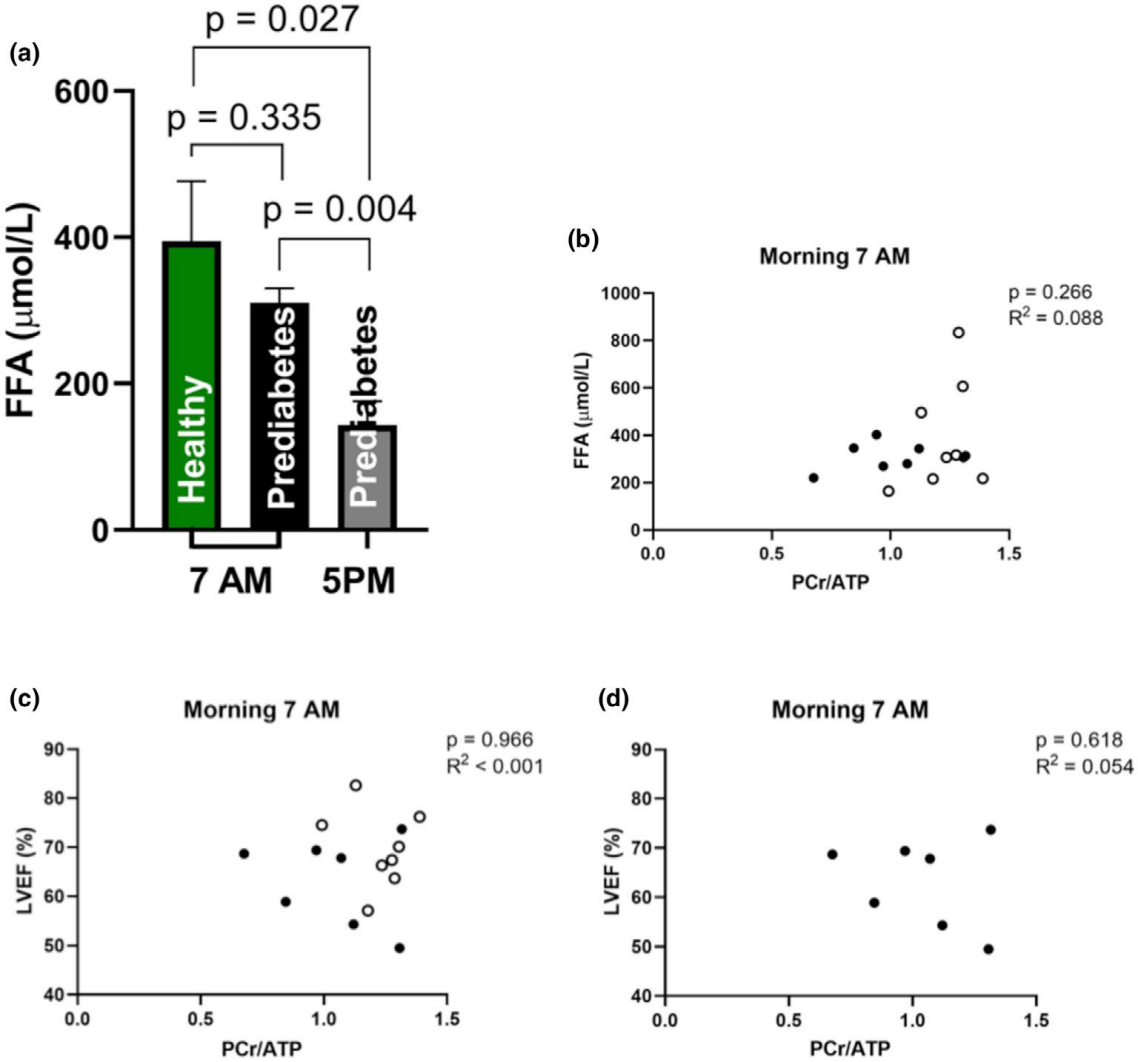
Plasma levels of FFA varied along the day and are not correlated to PCr/ATP and PCr/ATP ratio is not correlated to LVEF (%) in the fasted state. (a) Plasma levels of FFA were not different between the prediabetic and overweight and obese volunteers (*p* = 0.34), but they were significantly different between 7 am and 5 pm in the volunteers with prediabetes (*p* < 0.01). (b) PCr/ATP ratio and plasma levels of FFA at 7 am were not correlated (*p* = 0.27). (c and d) No correlation was found in the morning at 7 am in the healthy overweight and obese volunteers together with the volunteers with prediabetes (*n* = 15, panel c), neither in the volunteers with prediabetes (*n* = 7, panel d). Healthy overweight and obese volunteers are depicted as a white point, the volunteers with prediabetes are depicted as a black point. Values are depicted as mean ± standard error of the mean. The data of the healthy volunteer group were derived from the previous study of Remie et al. (2020) and used as a reference for the data in this study.

In the morning, the FFA levels from the volunteers with prediabetes did not differ significantly from the overweight and obese volunteers (Figure 3a, *p* = 0.335). Over the day FFA levels decreased in volunteers with prediabetes (Figure 3a, *p* < 0.01).

### 3.5 Cardiac PCr/ATP ratio is not correlated with plasma free fatty acids

Correlation analysis in the prediabetic and overweight/obese volunteers showed no relation between PCr/ATP ratio and FFA, as shown in Figure 3b (*p* = 0.27).

### 3.6 No day‐night fluctuations in cardiac systolic function

Left ventricular ejection fraction as measured with MRI was not significantly different between the overweight and volunteers with prediabetes, as shown in Figure 2c. Neither differences were found between morning and evening measurements in the volunteers with prediabetes (Figure 2c,d). The volunteers with prediabetes did have a significantly larger left ventricular volume during systole and diastole, as described in Table 2. However, according to the reference values, all volunteers had a normal LVEF and volume, also see Figure 2d and Table 2 (Kawel‐Boehm et al., 2015).

**TABLE 2:** Left ventricular volumes and ejection fraction.

		ESV (mL)	EDV (mL)	Stroke vol (mL)	LVEF (%)
Controls	7 AM	35.0 ± 14.5	119.4 ± 28.3	84.4 ± 19.3	71.2 ± 7.7
Prediabetes	7 AM	54.3 ± 19.4^a^	155.8 ± 29.6^a^	101.4 ± 16.1^a^	64.4 ± 9.0
	5 PM	57.3 ± 18.2	163.4 ± 30.1	106.1 ± 16.1	65.4 ± 5.4

*Note*: Values are depicted as mean ± standard deviation (*n* = 9). We significantly larger in the volunteers with prediabetes. The data of the healthy volunteer group were derived from the previous study of Remie et al. (2020) and used as a reference for the data in this study.

Abbreviations: EDV, end diastolic left ventricular volume; ESV, end systolic left ventricular volume; SV, stroke volume.

^a^ Indicates a significant difference between volunteers with prediabetes and control volunteers at 7 am. No significant differences were found between the measurements at 7 am and 5 pm in volunteers with prediabetes.

### 3.7 No correlation between cardiac PCr/ATP ratios and cardiac function

In seven volunteers with prediabetes and in eight overweight or obese volunteers, both PCr/ATP and LVEF were determined at 7 am. Correlation analysis in the whole group (*n* = 15, Figure 3c), as well as in the subgroup of volunteers with prediabetes (*n* = 7, Figure 3d), showed no relation between PCr/ATP ratio and LVEF. The delta between 7 am and 5 pm of PCr/ATP ratio was not correlated to the same delta of cardiac function.

## 4 DISCUSSION

Interestingly, the PCr/ATP ratio in the volunteers with prediabetes was lower in the morning, when compared to healthy overweight and obese controls. This suggests that the myocardial energy status is decreased already in prediabetes when LVEF is still normal. This supports the hypothesis that metabolic remodeling in prediabetic states proceeds functional and structural/geometrical remodeling of the heart regardless of the onset of overt hyperglycemia. This is in agreement with results from Perseghin et al. (2007) who showed that an abnormal cardiac energy status was detectable in obese men in the presence of normal function. Furthermore, the group of Rider et al. (2012) showed that cardiac energetics in obesity are further deranged during inotropic stress, in association with diastolic dysfunction. These data confirm that myocardial energetics may play a key role in the impairment of diastolic function in obesity and type 2 diabetes.

We hypothesized a lower myocardial energy status in prediabetes due to high circulating FFA, which stimulate the heart to rely more strongly on fat oxidation, which is less efficient in terms of ATP production relative to oxygen use. However, our results are not consistent with the hypothesis that the decrease in myocardial energy status is FFA dependent, since we did not find higher plasma FFA concentrations in the volunteers with prediabetes despite a lowered PCr/ATP. Also, we did not find a correlation between PCr/ATP and FFA levels. Furthermore, PCr/ATP was similar in the morning and afternoon, while FFA levels decreased from morning to afternoon. This suggests that FFA concentrations are not the main determinant of cardiac PCr/ATP. While some studies in the past did find negative correlations between PCr/ATP, cardiac function and FFA levels, this was not always the case. For example, Perseghin et al. also found no correlation (Perseghin et al., 2007), which is in agreement with the present study.

Alternatively, it has also been suggested that lowering of FFA, for example by the lipolysis inhibitor acipimox, reduces PCr/ATP (Watson et al., 2020). In line, pharmacological suppression of circulating FFA levels with Acipimox in patients with heart failure was associated with a detrimental effect on PCr/ATP ratio within few hours (Salerno et al., 2015), The same pharmacological modulation that affected PCr/ATP in heart failure patients was able to elicit the same response in healthy individuals in the short term (Lehto et al., 2012), The explanation for these effects probably is that FFA are an important source of energy for the heart and low FFA could be interpreted as an energy deprivation, thereby lowering cardiac energy status and subsequently PCr/ATP ratio. However, again our data can not confirm such a relation as the decrease in FFA from morning to afternoon did not decrease cardiac PCr/ATP. Probably, since substrate availability after a meal is high, lower FFA levels in the afternoon do not reflect energy deprivation.

Furthermore, it has been noted, that the fluctuations of plasma FFA during the day, associated with food intake, are tightly associated with changes in the circulating levels of ketone bodies (Ferrannini et al., 2016, 2022). Although ketone bodies during the day are relatively low, still, the changes in ketone bodies may also be of relevance and should also be taken in account when explaining these data. However, ketone bodies were not measured in this study and thus this issue remains unresolved.

Interestingly, when studying the relationship between cardiac energy status and cardiac function, we expected to find a functional relationship since a lower cardiac function may be associated with a lower cardiac energy status. However, in line with previous findings as reported in the study of Bilet et al. (2011), we did not find a relation between PCr/ATP ratio and LVEF in healthy volunteers. Hansch et al. did not find a relation between PCr/ATP ratio and LVEF in healthy volunteers neither, but did find a correlation in volunteers with severe and moderate dilated cardiomyopathy and a decreased LVEF (Hansch et al., 2005). This suggests that below a certain level of PCr/ATP ratio the LVEF may be negatively influenced, and it could be that in our insulin resistant prediabetic volunteers PCr/ATP ratio was still above this critical level.

Furthermore, no differences in cardiac LVEF in the morning versus evening measurements were found in the volunteers with prediabetes. In contrast, in literature, a lower LVEF was found in the morning upon waking in healthy volunteers compared to evening (Veale et al., 1994). Possibly this reduction in LVEF in healthy individuals is mediated by a greater change in plasma FFA over the day, whereas in our volunteers the change in FFA between morning and afternoon was relatively small. This is interesting, as it emphasizes the role of normal plasma metabolite oscillations in the maintenance of a normal cardiac cycle and nocturnal dipping, which apparently is absent in the prediabetic state.

We here show for the first time that PCr/ATP is not only decreased in patients with type 2 diabetes but already in a pre‐stage of actual diabetes. The decreased PCr/ATP in the prediabetic state is important in the context of cardiovascular disease (CVD), as the PCr/ATP ratio was shown to be a predictive value for CVD morbidity and mortality (Neubauer et al., 1997) and hence is an important marker of cardiovascular health. As this patient group is known to be at increased risk for CVD (Kannel et al., 1974; Punthakee et al., 2018; van de Weijer et al., 2011) which precedes the onset of T2DM, early interventions improving cardiac metabolism may improve their CVD risk profile and prevent the onset of cardiac disease.

## 5 LIMITATIONS

Although the number of volunteers was small, we found a lower PCr/ATP in the fasted state in volunteers with prediabetes in comparison to overweight or obese volunteers. For the comparison between the fasted and postprandial PCr/ATP ratio, the group was even smaller which bares the risk of missing small differences. Importantly, however, due to paired testing, power was sufficient to detect differences that are similar to the differences between the two groups. Including more volunteers probably would not have changed the results, as post hoc power analysis revealed an effect size of 0.08, which may be considered as a very small effect according to Cohen (1988) and therefore also a considerable (3–4 times) larger group size would not have resulted in a significant difference.

When comparing the groups it should be noted that the overweight or obese volunteers were slightly younger in comparison to the volunteers with prediabetes. Also, the volunteers with prediabetes were only males, the overweight or obese volunteers included also females. However, there is no literature claiming differences in PCr/ATP ratio between males and females.

## 6 CONCLUSION

We found a decreased myocardial energy status in prediabetes compared to healthy overweight or obese individuals, while the cardiac function remained normal. In addition, no fluctuations between morning and evening measurements of cardiac energy status and function in volunteers with prediabetes were found. As FFA did change considerably from the fasted to the fed state, and as FFA concentrations were not different between the two groups, this does not seem to be the underlying reason for lower PCr/ATP in prediabetes.

## AUTHOR CONTRIBUTIONS

VW, PS, VS, and TW designed research. VW, JW, and CR performed research and analyzed the data. VW, JW, CR, PS, VS, and TW reviewed data and performed data interpretation. VW wrote the paper. All authors reviewed the paper, gave input to improve the paper. All authors read and approved the final manuscript.

## FUNDING INFORMATION

Tineke van de Weijer was supported by a junior fellowship by the Dutch Diabetes Foundation (Grant No. 2015.81.1833) and Vera Schrauwen‐Hinderling was supported by a Grant from the European Research Council (ERC‐2017‐StG‐759161).

## CONFLICT OF INTEREST STATEMENT

The authors declare that they have no conflict of interests.

## ETHICS STATEMENT

The study was conducted in accordance with the principles of the declaration of Helsinki and approved by the Ethics Committee of the Maastricht University Medical Center.

## CONSENT TO PARTICIPATE

All volunteers provided written informed consent. The study was registered at https://clinicaltrials.gov with identifier NCT03733743 (07/11/2018).

## CONSENT FOR PUBLICATION

Consent for publication is given where applicable.

## Data Availability

The datasets used and/or analyzed during the current study are available from the corresponding author on reasonable request.
